# Correction to Circular RNA hsa_circ_001895 serves as a sponge of microRNA‐296‐5p to promote clear cell renal cell carcinoma progression by regulating SOX12

**DOI:** 10.1111/cas.15968

**Published:** 2023-10-02

Chen, Z, Xiao, K, Chen, S, Huang, Z, Ye, Y, Chen, T. Circular RNA hsa_circ_001895 serves as a sponge of microRNA‐296‐5p to promote clear cell renal cell carcinoma progression by regulating SOX12. Cancer Sci. 2020; 111: 713–726.

In this article, the author found that there was an error in Figure 7B.

The corrected Figure 7 is shown here: 
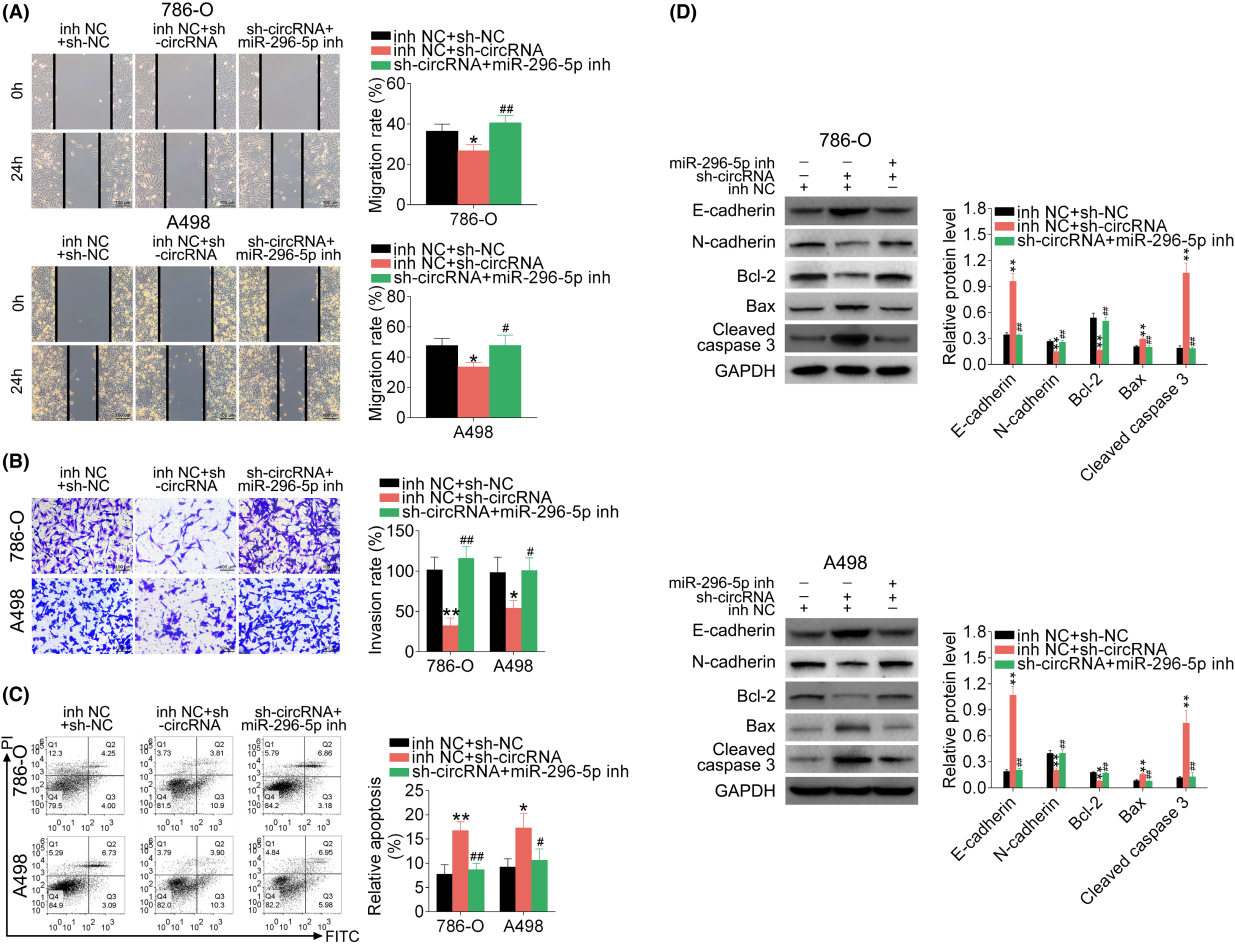


We apologize for this error.

